# Stress–Dilatancy Behaviourof Fibre-Reinforced Sand

**DOI:** 10.3390/ma16020609

**Published:** 2023-01-08

**Authors:** Zenon Szypcio, Katarzyna Dołżyk-Szypcio, Iwona Chmielewska

**Affiliations:** Department of Geotechnics and Structural Mechanics, Faculty of Civil Engineering and Environmental Sciences, Bialystok University of Technology, Wiejska 45E, 15-351 Bialystok, Poland

**Keywords:** sand, fibre reinforcement, stress–dilatancy

## Abstract

This paper analyses the stress–strain behaviour of fibre-reinforced sand using the results obtained by drained triaxial compression tests presented in the literature. The general stress–plastic dilatancy equation of the Frictional State Concept has been used to describe the behaviour of fibre-reinforced sand for different shear phases. The behaviour of pure sand is taken as a reference for the behaviour of sand with added fibres. It is shown that the characteristic shear phases can only be determined when the $\eta -D^{p}$ relationships are used, which are very rarely demonstrated in the results of shear tests presented in the literature. It has been shown that tensile strains must occur in order to achieve the strengthening effect of fibre reinforcement. A reduction in the stiffness of the fibre–sand composite is observed in the absence of tensile strains below certain threshold values.

## 1. Introduction

Using natural fibres to reinforce weak soils is an old and ancient idea [1]. Natural fibres include coconut, sisal, palm, jute, bamboo, straw, and others [2]. Synthetic fibres include polypropylene, polyester, polyethylene, glass, and nylon [2]. The tensile strength of natural and synthetic fibres is higher than that of many soils. Therefore, fibres randomly distributed in the soil can increase the tensile and shear strengths of the fibre–soil composite. The effect of fibre reinforcement not only depends on the type and conditions of the soil and the concentration of fibres within it but also on the fibres’properties, orientation, lengths, and the interactions between the fibres and the soil [3]. In recent years, the fibre-reinforcement technique has been successfully used to stabilise slopes, road subgrades, and embankments [1,2,3,4]. The macroscopic material properties of fibre-reinforced soils have been studied in the laboratory by many researchers [5,6,7,8,9]. Additionally, numerical DEM simulations can both quantify these macro-properties as well as the mechanical fibre–soil interactions [2,10,11,12]. Zornberg [13], for example, analysed the limit equilibrium of soil using the discrete framework.

Many fibre-reinforced soil models have been developed to describe behaviour at various initial states, stress levels, and stress paths [14,15,16,17].

The stress–dilatancy relationship is commonly used as the basis for the development of constitutive models for soils [18,19,20]. The best known are the stress–dilatancy relationships of Rowe [21] and Bolton [22]. These stress–dilatancy relationships are correct for granular soils with high single grain strength. These relationships need to be modified for specific soils, such as coral sand [23,24] or fibre-reinforced sand [25,26]. New density- and stress level-dependent parameters must be introduced into the classical stress–dilatancy relationships. The general stress–dilatancy relationship was developed by Szypcio [27] based on the Frictional State Concept (FSC). This relationship is determined by the critical frictional state angle ($\phi^{o}$) and two soil parameters α and β. For drained triaxial compression failure states of sand, failure states are represented by points on the $\eta -D^{p}$ plane lying on the frictional state line (FSL) with α=0 and β=1 [28]. The analysis of the stress–plastic dilatancy relationship for drained triaxial compression of railway ballast [29] and limestone gravel [30] shows that in different shearing phases, they can be well approximated by straight lines described by the general stress–plastic dilatancy FSC relationship. The points representing the failure states lie on a straight line with a greater slope than for pure sand due to the breakage effect. For artificially cemented sand, the states of minimum dilatancy, called dilatant failure states, do not coincide with the failure states, which are states of maximum stress ratio ${\left({ο}_{1}^{\prime }/\sigma_{3}^{\prime }\right)}_{max}$ [31]. The points representing the dilatant failure states lie on a straight line called the dilatant failure state line (DFSL). The location and the slope of the dilatant failure state line in the $\eta -D^{p}$ plane are independent on the density and stress levels [31]. Note that the original and modernised Rowe and Bolton stress–dilatancy relationships, unlike the general FSC stress-plastic dilatancy relationship, cannot describe the actual shear behaviour of soils at all shear phases.

In this paper, the results of drained triaxial compression tests of various fibre-reinforced sands published in the geotechnical literature will be analysed. The stress–dilatancy relationship of these fibre-reinforced sands will be calculated and compared with the relationship of a soil sample without any additional fibres. The influence of the sample preparation method on this relationship will also be analysed.

## 2. The General Stress–Plastic Dilatancy Relationship

The general Stress–Plastic dilatancy relationship adopted in this paper is based on the Frictional State Concept and has the following form [27]:

(1) $$\eta =Q-AD^{p}$$

where

(2) η=q/p′

(3) $$D^{p}=\delta \varepsilon_{\upsilon }^{p}/\delta \varepsilon_{q}^{p}$$

(4) $$Q=M^{o}-\alpha A^{o}$$

(5) $$A=\beta A^{o}.$$

For triaxial compression:

(6) $$M^{o}=M_{c}^{o}=6\;sin\phi^{o}/\left(3-sin\phi^{o}\right)$$

(7) $$A^{o}=A_{c}^{o}=1-\frac{1}{3}M_{c}^{o}$$

where $\phi^{o}$ is the critical frictional state angle. For sands, $\phi^{o}=\phi_{c\upsilon }^{\prime }$ [28].

The parameters α and β are new parameters of the Frictional State Concept [27]. The shear state for which α= 0 and β= 1 is a purely frictional state. Thus, α and (β−1) represent the deviation of the current shear state from the purely frictional state.

The plastic parts of the volumetric and shear strain increments are calculated from the following equations:

(8) $$\delta \varepsilon_{\upsilon }^{p}=\delta \varepsilon_{\upsilon }-\frac{\delta p\prime }{Κ}$$

(9) $$\delta \varepsilon_{q}^{p}=\delta \varepsilon_{q}-\frac{\delta q}{3G}$$

(10) Κ=2G(1+v)/{3(1−2v)}

where *Κ* and *G* are the elastic bulk and shear modulus, v is the Poisson’s ratio, and $p^{\prime }=\left(\sigma_{1}^{\prime }+2\sigma_{3}^{\prime }\right)/3$, $q=\sigma_{1}^{\prime }-\sigma_{3}^{\prime }$, $\varepsilon_{\upsilon }=\varepsilon_{1}+2\varepsilon_{3}$, $\varepsilon_{q}=2\left(\varepsilon_{1}-\varepsilon_{3}\right)/3$, $\varepsilon_{1}=\varepsilon_{a}$.

For sand, it is usually assumed that

(11) $$G=G_{0}\frac{{\left(2.97-e\right)}^{2}}{1+e}\sqrt{p\prime p_{a}}$$

where $G_{0}$ is the sand parameter and $p_{a}=101\;\mathrm{kPa}$ and is atmospheric pressure.

## 3. Methodology

The relationships $q-\varepsilon_{a}$ and $\varepsilon_{\upsilon }-\varepsilon_{a}$ presented in the literature, obtained from drained triaxial compression tests, are segmentally approximated using high-degree polynomials. The stress ratio (η), the plastic parts of the volumetric strain increments ($\delta \varepsilon_{\upsilon }^{p}$), the plastic parts of the shear strain increments ($\delta \varepsilon_{q}^{p}$), and the plastic dilatancy ($D^{p}$) were calculated in this paper using these polynomials. The relationships between the stress ratio and the plastic dilatancy were calculated and presented in the figures for all the tests analysed.

The authors focused particular attention to the connection points on the approximated segments of the analysed $\varepsilon_{\upsilon }-\varepsilon_{a}$ relationship. At the segments’ connection point, the values of volumetric strain ($\varepsilon_{\upsilon }$) and the strain increment ratios ($\delta \varepsilon_{\upsilon }/\delta \varepsilon_{a}$) should be the same. In the $\eta -D^{p}$ relationships, smoothness disturbances are visible if these conditions are not fulfilled.

## 4. Stress–Plastic Dilatancy Relationships for Sand with Polyamide Fibres

### 4.1. Analysed Tests

Michalowski and Čermák [5] conducted a series of drained triaxial compression tests to study the behaviour of fine- and medium-grained sands reinforced with different fibre types. The polyamide fibres used in the tests had a length of 25.4 mm and an aspect ratio (length/diameter) of 85. For comparison, tests with pure fine sand were also performed. Drained triaxial tests were carried out on cylindrical samples with a height and diameter of 94.5 mm. All samples of unreinforced and reinforced sand were made at the initial void ratio $e_{0}=$ 0.58, which corresponds to the relative density of unreinforced sand, $I_{D}=$ 70%. A special sample preparation technique was adopted to obtain samples with a uniform distribution of fibres in space and a uniform distribution of fibre orientations in all directions [5]. Compared to the behaviour of pure fine sand, the addition of fibres reduces dilatancy and initial stiffness and increases shear strength. These effects are a function of initial density, fibre concentration, strength, the length and aspect ratio of the fibres, and the stress level.

In the presented paper, three tests of fine sand with a content of 0.5% and three tests with a volume content of 2% of polyamide fibres will be performed, and all the tests of pure sand performed by Michałowski and Čermák [5] will be analysed.

### 4.2. Characteristic Stages of Shearing

**Figure 1:**
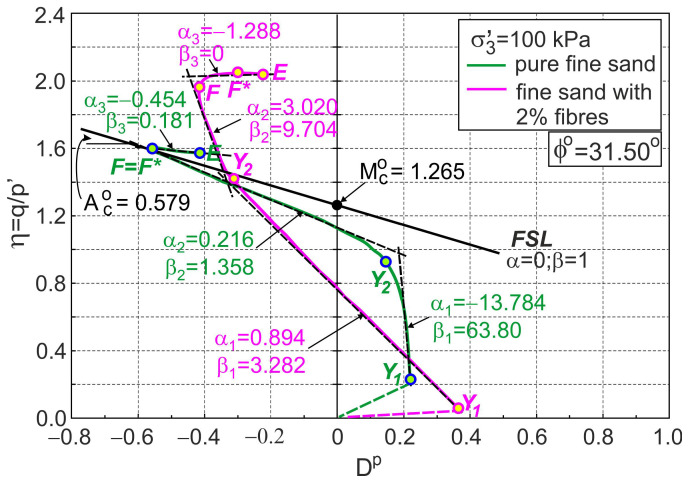
Characteristic stages of shearing of pure and fibre-reinforced fine sand (based on the experimental data from [5]).

Four characteristic shear stages can be observed during drained triaxial compression tests of pure sand [31], railway ballast [29], limestone gravel [30], artificially bonded soils [32], and fibre-reinforced sand (Figure 1). The elasticity stage occurs when the behaviour of the material is elastic. The end of this stage is marked as point $Y_{1}$ in Figure 1. The elastic stage is followed by three elasto-plastic stages. During the first elasto-plastic stage, the elastic parts of the strain increments in the global strain increments rapidly decrease, and the rate of decrease of the tangent shear modulus grows [32]. The end of this stage is marked as point $Y_{2}$ in Figure 1. During the second elasto-plastic stage, the tangent shear modulus values decrease to zero. The end of this stage is called the dilatant failure state and is marked as point F in Figure 1.

The dilatant failure state is defined by the minimum plastic dilatancy (Figure 1). The third elasto-plastic stage of shearing is called the post-dilatant failure stage. At this stage of shearing, the decrease in the stress ratio with increase in plastic dilatancy is observed for drained triaxial compression of pure sand or other granular materials [32]. For granular materials, dilatant failure states are equivalent to classical failure states [29,30,31]. In Figure 1, these stages are shown for fine sand with 2% polyamide fibres. The dilatant failure states (F) and classical failure states (F*) are not equivalent for the fibre-reinforced sands as they are for artificially cemented soils [32].

For all elasto-plastic stages, the relationship between the stress ratio and the plastic dilatancy can be approximated by straight lines, given by Equation (1). In this paper, pure sand is assumed as a reference material, and Equation (1) is defined by the critical frictional state angle of pure sand $\phi^{o}(M_{c}^{o}\;\mathrm{and}\;A_{c}^{o}$) and the appropriate values of $\alpha_{i}$ and $\beta_{i}$ for various stages of shearing pure sand and fibre-reinforced sand (Figure 1). For pure sand, the dilatant failure state is represented by a point on the frictional state line (FSL) where α= 0 and β= 1 in the $\eta -D^{p}$ plane [31]. For the sample of pure fine sand tested by Michalowski and Čermák [5], $\phi^{o}=$ 31.5°, $M_{c}^{o}=$ 1.265, and $A_{c}^{o}=$ 0.579 (Figure 1).

### 4.3. Stress–Strain Behaviour of Pure Fine Sand

**Figure 2:**
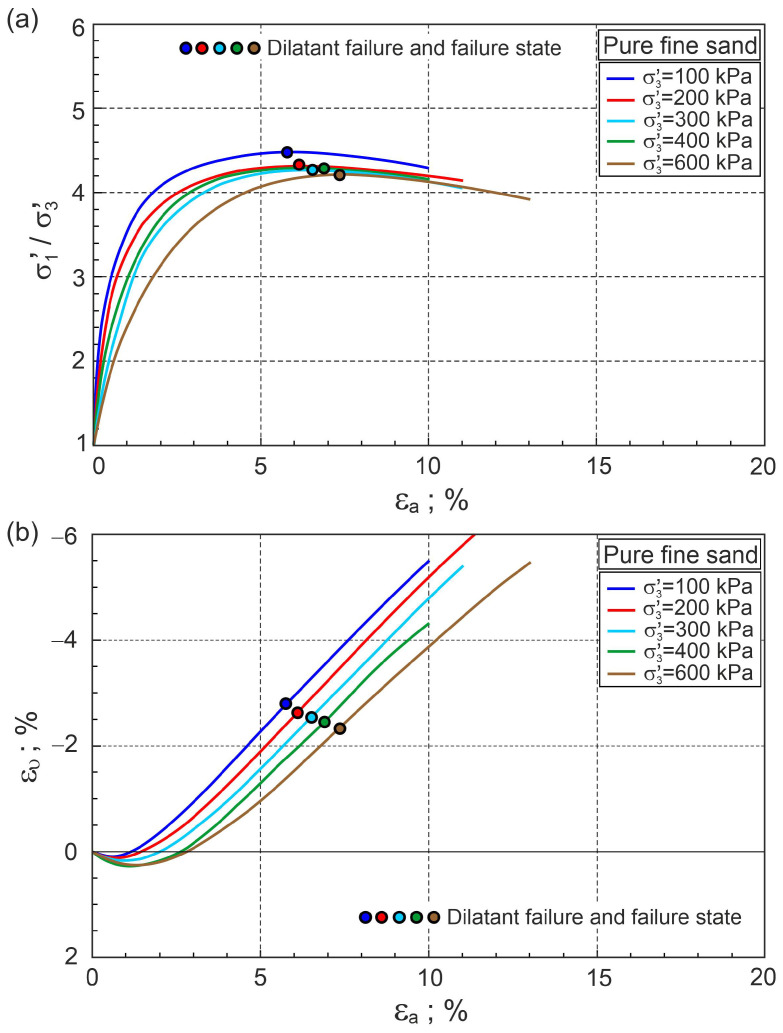
Relationships for pure fine sand: (**a**) $\sigma_{1}^{\prime }/\sigma_{3}^{\prime }-\varepsilon_{a}$ and (**b**) $\varepsilon_{\upsilon }-\varepsilon_{a}$ (experimental data from [5]).

**Figure 3:**
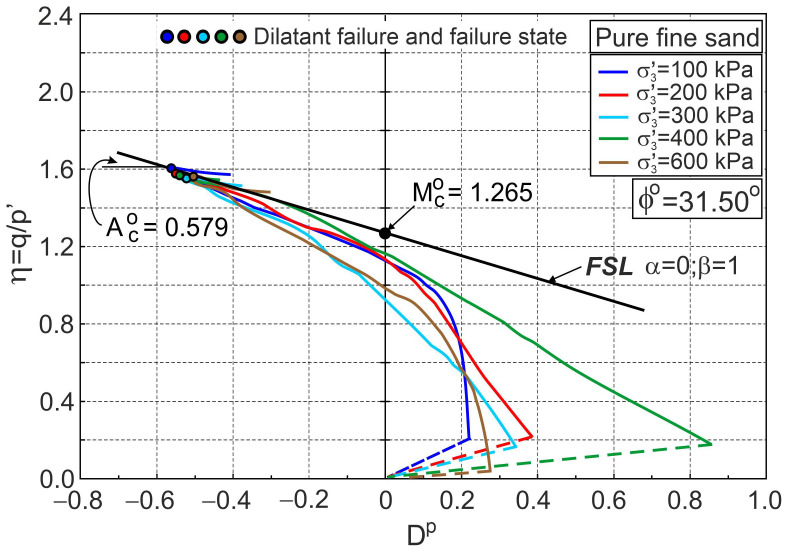
Stress ratio–plastic dilatancy relationships for pure sand (based on the experimental data from [5]).

Figure 2 shows the $\sigma_{1}^{\prime }/\sigma_{3}^{\prime }-\varepsilon_{a}$ and $\varepsilon_{\upsilon }-\varepsilon_{a}$ relationships for a pure sand sample tested by Michalowski and Čermák [5]. The stress ratio–plastic dilatancy relationships are shown in Figure 3.

In Figure 2 and Figure 3, only dilatant failures equivalent to failure states are marked for clarity. The points representing dilatant failure states lie almost exactly on the frictional state line (FSL) (Figure 3). The characteristic shear stages are evident for all tests. In the calculations, the elastic parameters $G_{0}=$ 120 and v= 0.3 and the initial void ratio $e_{0}=$ 0.58 were accepted. For many drained triaxial tests of sands in the post-dilatant failure stage, the stress ratio–plastic dilatancy relationship is represented by the frictional state line [31,33]. This is not fully confirmed for the tests on pure fine sand analysed here (Figure 3). The source of these differences is probably the low slenderness (*H*/*D* = 1) of the tested samples. Characteristic shear stages are only visible in the $\eta -D^{p}$ relationships, not in the $\sigma_{1}^{\prime }/\sigma_{3}^{\prime }-\varepsilon_{a}$ and $\varepsilon_{\upsilon }-\varepsilon_{a}$ relationships (Figure 2 and Figure 3). Unfortunately, $\eta -D^{p}$ relationships are presented in less than 0.5% of published test results.

### 4.4. Stress–Strain Behaviour of Fibre-Reinforced Sand

**Figure 4:**
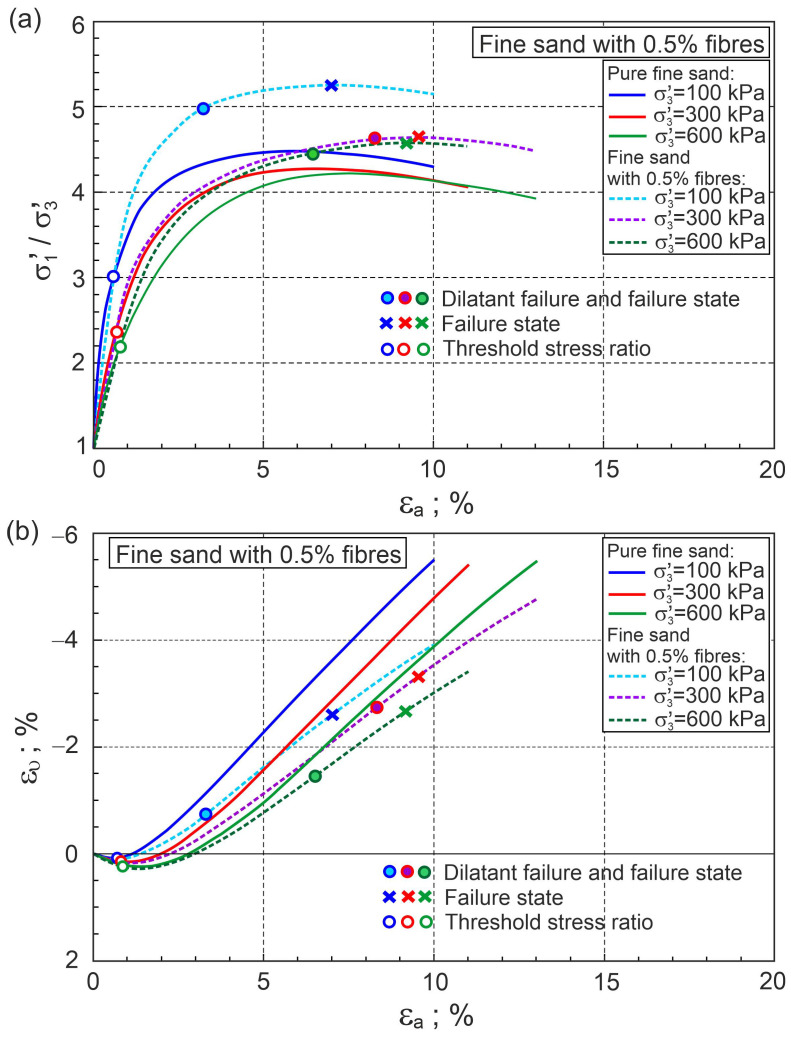
Relationships for fine sand with 0.5% fibres: (**a**) $\sigma_{1}^{\prime }/\sigma_{3}^{\prime }-\varepsilon_{a}$ and (**b**) $\varepsilon_{\upsilon }-\varepsilon_{a}$ (experimental data from [5]).

**Figure 5:**
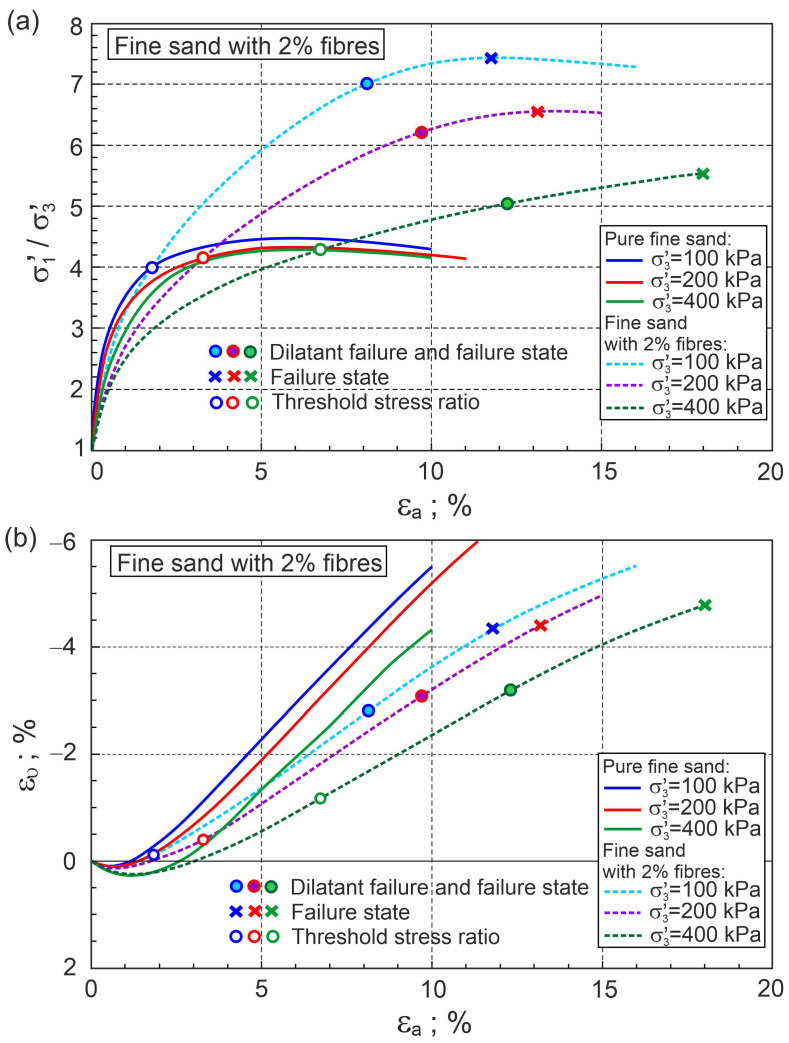
Relationships for fine sand with 2% fibres: (**a**) $\sigma_{1}^{\prime }/\sigma_{3}^{\prime }-\varepsilon_{a}$ and (**b**) $\varepsilon_{\upsilon }-\varepsilon_{a}$ (experimental data [5]).

The ratios of principal stresses to axial strain and volume strain to axial strain for fine sand with a volume content of fibres of 0.5% and 2% are shown in Figure 4 and Figure 5, respectively.

**Figure 6:**
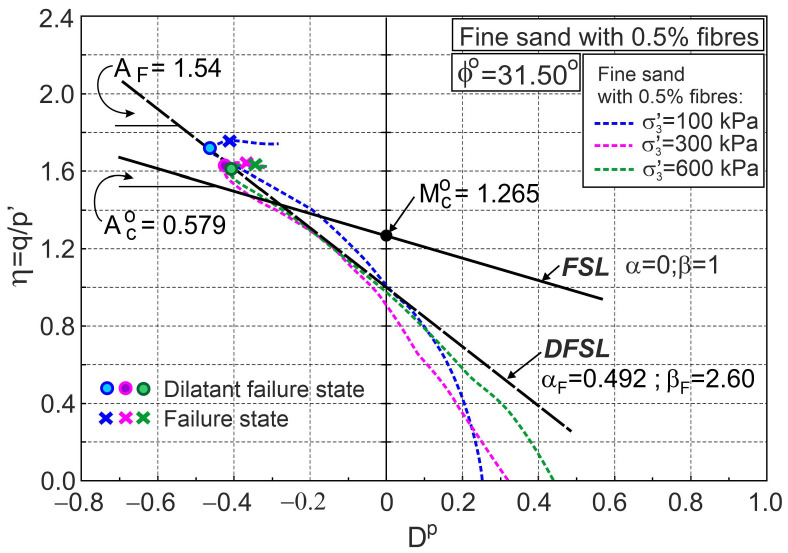
Stress ratio–plastic dilatancy relationships for sand with 0.5% fibres (based on the experimental data from [5]).

**Figure 7:**
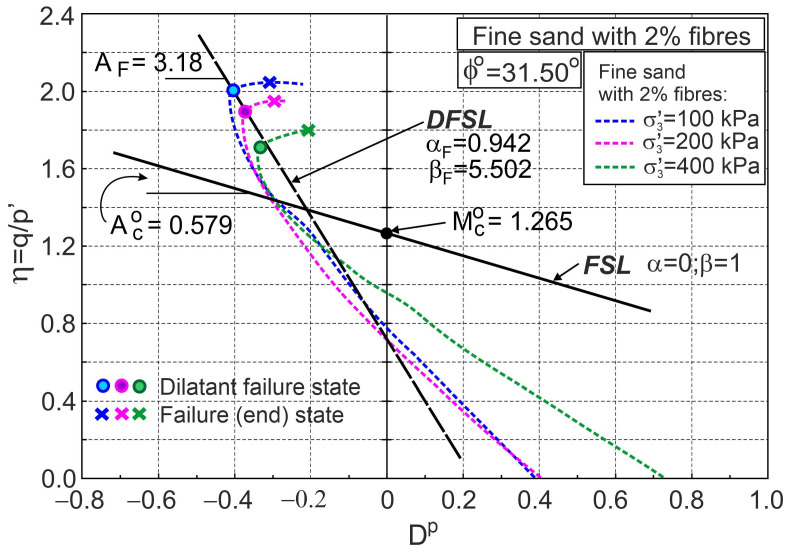
Stress ratio–plastic dilatancy relationships for sand with 2% fibres (based on the experimental data from [5]).

Figure 4 and Figure 5 also show similar relationships for pure fine sand for comparison. The addition of fibres significantly reduces volumetric expansion and increases shear strength. In the first elasto-plastic stage, the lines representing the principal stress ratio versus axial strain for pure sand are above the corresponding lines for fibre-reinforced sand (Figure 4a and Figure 5a). This means that the stiffness of the fibre-reinforced sand is lower than that of pure sand in this shear phase. States for which the principal stress ratios for pure sand and sand with fibres are equal at the same confining pressure are called threshold principal stress ratios. The threshold principal stress ratio is a function of fibre concentration and stress level (Figure 4a and Figure 5a). Relationships between the stress ratio and plastic dilatancy for fine sand with 0.5% and 2% fibre contents are shown in Figure 6 and Figure 7, respectively.

Calculations were made for v= 0.3 and $G_{0}=$ 100 and $G_{0}=$ 80 for sand with 0.5% and 2% volumetric fibre contents, respectively.

**Figure 8:**
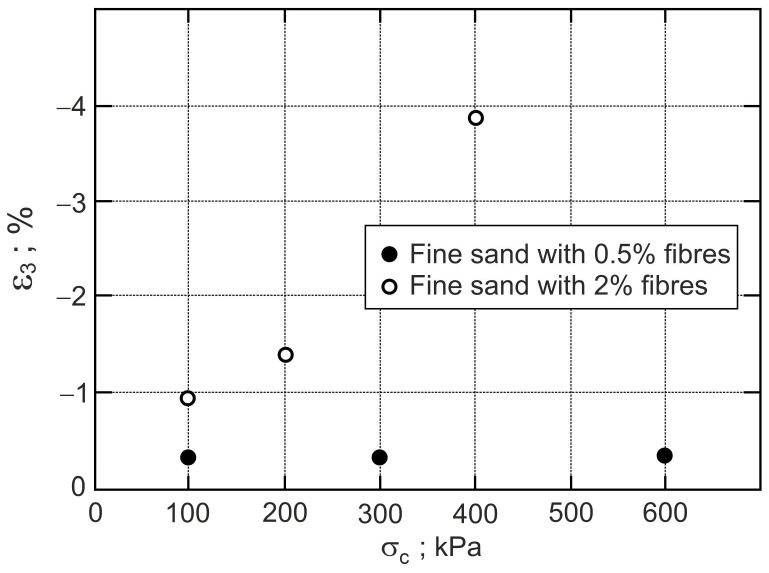
Principal tensile strain at threshold states.

The points that represent dilatant failure states lie on a straight dilatant failure state line (DFSL) defined by $\phi^{o}=$ 31.5° and $\alpha_{F}=$ 0.452, $\beta_{F}=$ 2.60 for sand with a 0.5% fibre content (Figure 6), and $\alpha_{F}=$ −0.942, $\beta_{F}=$ 5.502 for a 2% fibre content (Figure 7). The higher the fibre content, the greater the slope of the dilatant failure state line. Characteristic states of behaviour of sand reinforced with fibres are shown in Figure 4, Figure 5, Figure 6 and Figure 7. The fibres only strengthen the sand when they are extended. Therefore, the maximum reinforced effect results from the fibres being arranged parallel to the direction of maximum extension ($\varepsilon_{3}$). Fibres that deviate from these directions only partially influence the strengthening and stiffness of the sand–fibre composite. Fibres arranged parallel to the contraction direction do not have a reinforcing effect and reduce the stiffness of the sand–fibre composite [16]. The values of the principal tensile strains for which the fibres give a reinforcing effect for the analysed sand–fibre composite are shown in Figure 8. The effect of fibre reinforcement can manifest itself not only in the dilative (Figure 5) but also in the contractive (Figure 4) behaviour of the sand with fibres during shear. It is practically possible to arrange the fibres evenly in the soil. The use of fibres to reduce the expansibility of clay is very effective as, in all directions, tensile stresses are potentially induced in the fibres. The effect of the fibres on strength is greater in triaxial compression with two principal tension directions than in triaxial extension with only one principal tension direction [16].

The effectiveness of fibre reinforcement using the same sand and fibres depends on the concentration, length, and strength of the fibres, as well as the stress level. This should be taken into account when solving each geotechnical problem.

## 5. Effect of Sample Preparation Methods

**Figure 9:**
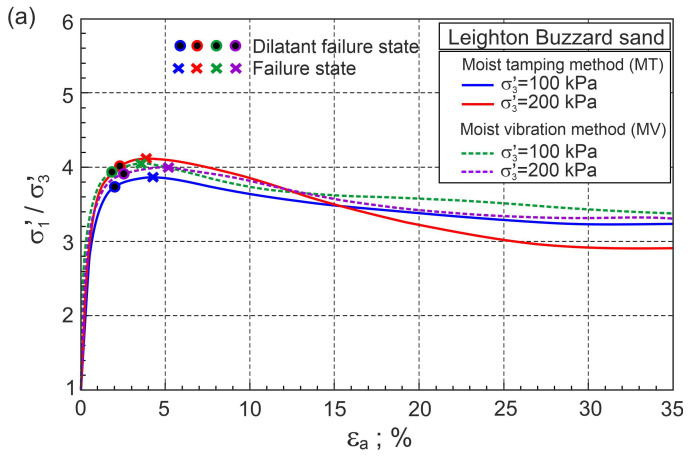

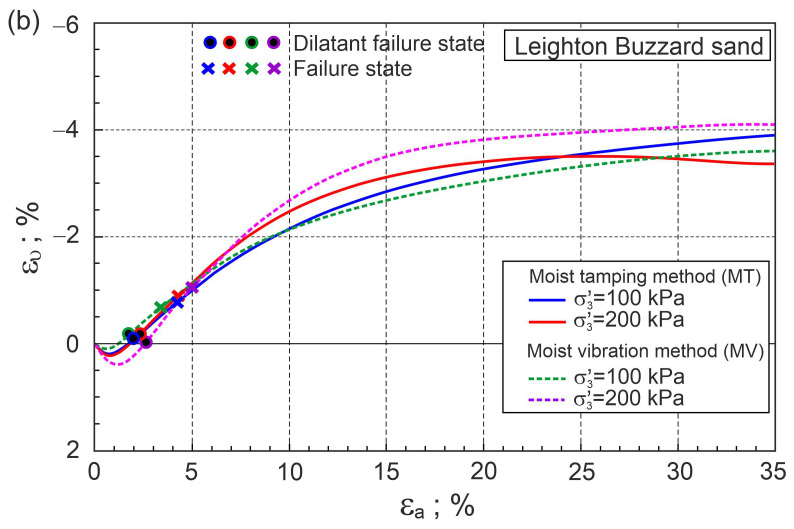
Relationships for pure Leighton Buzzard sand: (**a**) $\sigma_{1}^{\prime }/\sigma_{3}^{\prime }-\varepsilon_{a}$ and (**b**) $\varepsilon_{\upsilon }-\varepsilon_{a}$ (experimental data from [34]).

The method of preparation of the sample may affect the fibre arrangement and homogeneity of the prepared samples. The influence of the sample preparation method on the stress–strain behaviour of pure Leighton Buzzard sand and sand with 0.25% by weight of fibres was studied by Gao and Huang [34]. Moist tamping (MT) and moist vibration (MV) methods were used. In this paper, only selected experimental results of drained triaxial compression tests on samples with initial void ratios of $e_{0}=$ 0.81 and confining pressures $\sigma_{3}^{\prime }=$ 100 kPa and 200 kPa were analysed. The principal stress ratios ($\sigma_{1}^{\prime }/\sigma_{3}^{\prime }$) and volumetric strains ($\varepsilon_{\upsilon }$) as functions of axial strain ($\varepsilon_{a}$) for pure Leighton Buzzard sand are shown in Figure 9.

**Figure 10:**
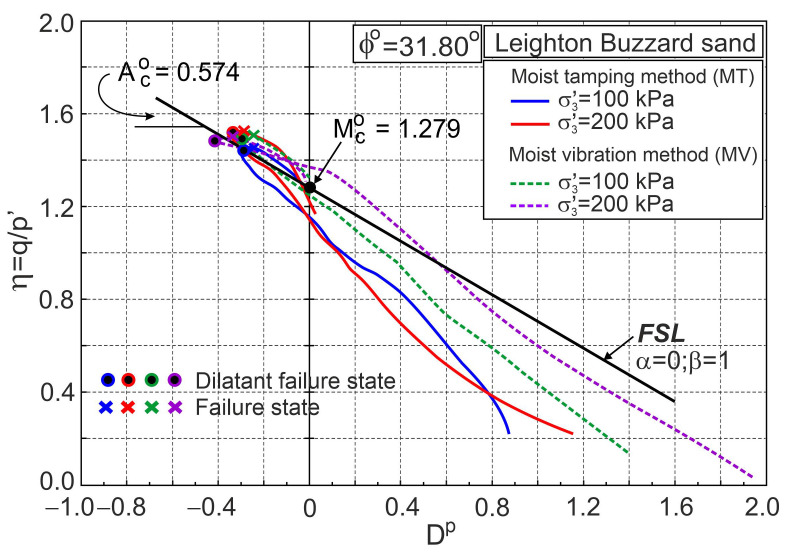
Stress ratio–plastic dilatancy relationships for pure Leighton Buzzard sand (based on the experimental data from [34]).

The stress ratio–plastic dilatancy relationships for pure Leighton Buzzard sand are shown in Figure 10. Dilatant failure states and failure states are marked in Figure 9 and Figure 10, respectively. The dilatant failure states and failure states do not coincide with the results for pure fine sand tested by Michalowski and Čermák [5]. The differences do not seem significant, but they are. All points representing dilatant failure states lie almost on the straight frictional state line (FSL) drawn for $\phi^{o}=$ 31.8° (Figure 9). The method of sample preparation has no significant influence on the behaviour of pure Leighton Buzzard sand during shear [34].

**Figure 11:**
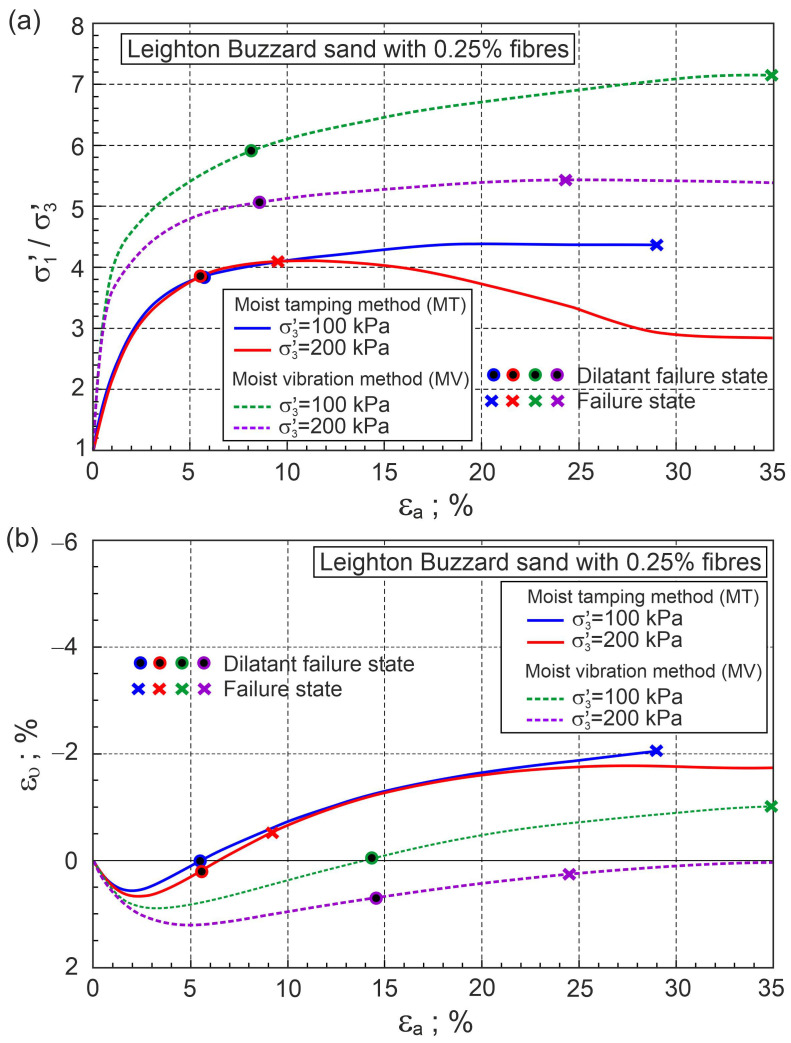
Relationships for Leighton Buzzard sand with 0.25% of fibres: (**a**) $\sigma_{1}^{\prime }/\sigma_{3}^{\prime }-\varepsilon_{a}$ and (**b**) $\varepsilon_{\upsilon }-\varepsilon_{a}$ (experimental data from [34]).

**Figure 12:**
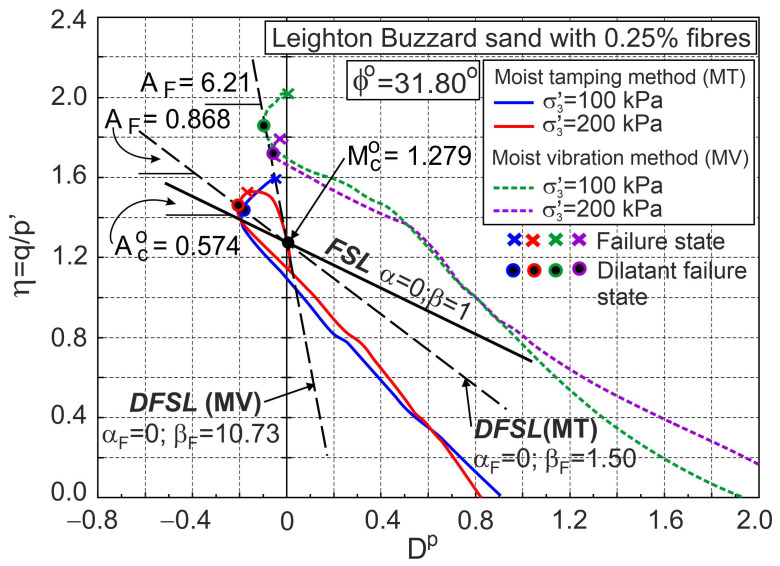
Stress ratio–plastic dilatancy relationships for Leighton Buzzard sand with 0.25% of fibres (based on the experimental data from [34]).

Similarly, the relationships $\sigma_{1}^{\prime }/\sigma_{3}^{\prime }-\varepsilon_{a}$, $\varepsilon_{\upsilon }-\varepsilon_{a}$, and $\eta -D^{p}$ for drained triaxial compression of Leighton Buzzard sand with 0.25% of fibres are shown in Figure 11 and Figure 12.

It can be seen that the method of sample preparation dramatically changes the behaviour of sand with fibres during shear. It can be assumed that the dilatant failure state lines for MT and MV sample preparation methods intersect the vertical axis at $\eta =M_{c}^{o}=$ 1.279 ($Q=M_{c}^{o}$ and $\alpha_{F}=0$). The slope of the dilatant failure line for the sample prepared with the MT method is $A_{F}=$ 0.868 ($\beta_{F}=$ 1.50) and the slope is $A_{F}=$ 6.21 ($\beta_{F}=$ 10.73) for the MV method. The difference between dilatant failure states is significantly dependent on the stress level (Figure 12). The relationship $\eta -D^{p}$ is helpful in fully describing the behaviour of pure sand and sand with fibres during shear.

## 6. Conclusions

(1) Only a few of the triaxial compression test results presented in the literature for two sands with different fibres were analysed, and it is possible to formulate only general conclusions;
(1) The general stress–plastic dilatancy ($\eta -D^{p}$) equation of the Frictional State Concept can correctly approximate the behaviour of fibre-reinforcement sand in different shear stages;
(3) The behaviour of pure sand can be taken as a reference for the behaviour of sand with fibres;
(4) The dilatant failure states of fiber-reinforced sand and artificially cemented soils differ. Dilatant failure states represent the more characteristic stress–plastic dilatancy behaviour;
(5) Fibre concentration, stress level, and the method of sample preparation significantly affect the behaviour of fibre–sand composites during shear;
(6) Tensile strains must be present in order to produce a reinforced effect in a fibre–sand composite. A reduction in the stiffness of the fibre sand composite is observed in the absence of tensile strains below a certain threshold value.

## Data Availability

Not applicable.
